# SB203580—A Potent p38 MAPK Inhibitor Reduces the Profibrotic Bronchial Fibroblasts Transition Associated with Asthma

**DOI:** 10.3390/ijms222312790

**Published:** 2021-11-26

**Authors:** Milena Paw, Dawid Wnuk, Kinga Nit, Sylwia Bobis-Wozowicz, Rafał Szychowski, Alicja Ślusarczyk, Zbigniew Madeja, Marta Michalik

**Affiliations:** Department of Cell Biology, Faculty of Biochemistry, Biophysics and Biotechnology, Jagiellonian University, Gronostajowa 7, 30-387 Kraków, Poland; dawid.wnuk@uj.edu.pl (D.W.); kinga.polanska@doctoral.uj.edu.pl (K.N.); sylwia.bobis@uj.edu.pl (S.B.-W.); raf.szychowski@gmail.com (R.S.); alicja.slusarczyk.97@wp.pl (A.Ś.); z.madeja@uj.edu.pl (Z.M.); marta.michalik@uj.edu.pl (M.M.)

**Keywords:** p38, MAPK, TGF-β_1_, fibroblast-to-myofibroblast transition, subepithelial fibrosis, myofibroblasts, asthma

## Abstract

Subepithelial fibrosis is a component of the remodeling observed in the bronchial wall of patients diagnosed with asthma. In this process, human bronchial fibroblasts (HBFs) drive the fibroblast-to-myofibroblast transition (FMT) in response to transforming growth factor-β_1_ (TGF-β_1_), which activates the canonical Smad-dependent signaling. However, the pleiotropic properties of TGF-β_1_ also promote the activation of non-canonical signaling pathways which can affect the FMT. In this study we investigated the effect of p38 mitogen-activated protein kinase (MAPK) inhibition by SB203580 on the FMT potential of HBFs derived from asthmatic patients using immunocytofluorescence, real-time PCR and Western blotting methods. Our results demonstrate for the first time the strong effect of p38 MAPK inhibition on the TGF-β_1_-induced FMT potential throughout the strong attenuation of myofibroblast-related markers: α-smooth muscle actin (α-SMA), collagen I, fibronectin and connexin 43 in HBFs. We suggest the pleiotropic mechanism of SB203580 on FMT impairment in HBF populations by the diminishing of TGF-β/Smad signaling activation and disturbances in the actin cytoskeleton architecture along with the maturation of focal adhesion sites. These observations justify future research on the role of p38 kinase in FMT efficiency and bronchial wall remodeling in asthma.

## 1. Introduction

Symptoms characteristic of bronchial asthma are the consequences of chronic inflammation observed in airway tracts and bronchial airway remodeling, which is a complex process leading to the thickening of the bronchial airway wall and airflow restriction [1]. The functional impairment of the interactions between the epithelial layer and deeper parts of bronchi (subepithelial layer with fibroblasts) within the epithelial-mesenchymal trophic unit [2,3,4,5] leads to the progression of subepithelial fibrosis. A key event of this process is the fibroblast-to-myofibroblast transition (FMT) induced by profibrotic and proinflammatory cytokines and growth factors, mainly transforming growth factor-β (TGF-β) [6]. Exposition of human bronchial fibroblasts (HBFs) to TGF-β leading to the constitutive activity of myofibroblasts is also responsible for the overproduction of extracellular matrix (ECM) proteins [1,6]. The enhanced levels of α-smooth muscle actin (α-SMA) incorporated into microfilaments anchored in super mature focal adhesion (FA) sites [6] are observed in myofibroblasts. Our earlier studies show that the FMT potential is enhanced in HBF populations derived from asthmatic (AS) donors in comparison to non-asthmatic (NA) ones [7,8,9,10,11]. This phenomenon is dependent on the activity of the canonical profibrotic TGF-β/Smad2/3 signaling pathway and closely associated with the function of connexin 43 [9,10,11,12]. Our recent report indicates that the disturbed balance between the activity of TGF-β_1_-stimulated profibrotic Smad2/3 and antifibrotic Smad1/5/9 pathways supports the enhanced FMT potential observed in HBF AS populations [10]. However, the role of non-canonical TGF-β_1_-induced signaling, especially MAPK (mitogen-activated protein kinase)-dependent pathways in asthma-related FMT remains insufficiently investigated. MAPK signaling includes three well-known pathways dependent on the activity of classical MAPK (also known as extracellular-signal regulated kinase; ERK), c-Jun N-terminal kinase/stress-activated protein kinase (JNK/SAPK) and p38 kinase [13]. Several reports indicate the involvement of ERK- and c-Jun-dependent MAPK signaling in the progression of liver [14,15], kidney [16,17], cardiac [18,19] and lung fibrosis [20,21]. The role of p38 MAPK was described in the fibrosis-related processes observed in the liver [22], kidneys [23,24], heart [25,26] and lungs [27]. Numerous reports indicate increased levels of p38 observed in sputum and bronchial tissue derived from asthmatic patients [28,29,30]. However, little is known about the role of p38 MAPK-dependent signaling in the effectiveness of phenotypic shifts of TGF-β_1_-treated HBFs derived from asthmatic patients. To fill this gap, in this study we investigated the effect of p38 MAPK inhibition on the efficiency of TGF-β_1_-induced FMT in the HBF AS populations.

## 2. Results

### 2.1. The TGF-β_1_-Induced Myofibroblastic Transition of HBFs AS Is Diminished in Response to the Administration of the p38 MAPK Inhibitor

**Figure 1:**
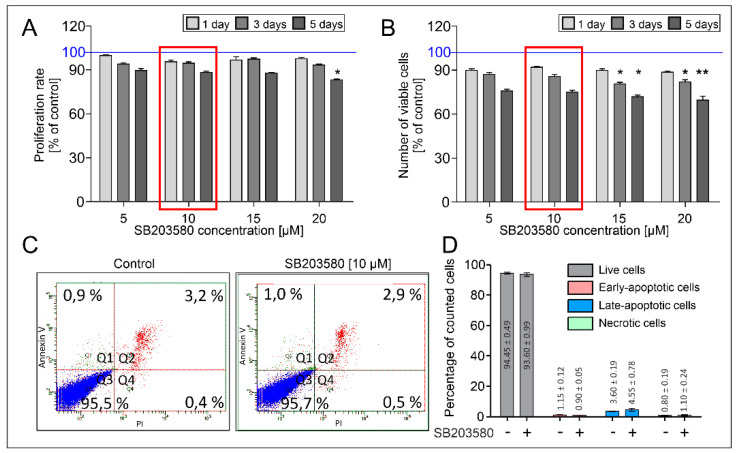
Low doses of SB203580 have a negligible effect on the proliferation, viability and apoptosis of HBFs AS. HBF populations (*n* = 3) were exposed to increasing concentrations (5–20 μM) of SB203580 for one, three and five days, each in triplicates. (**A**) The proliferation rate of HBFs measured by crystal violet assay. (**B**) The viability of HBFs determined with the MTT test. Results were presented as a percent of control cells cultured in serum-free medium without SB203580 exposition by one, three or five days. The percentage of apoptotic cells in HBF populations (30,000 events) treated by SB203580 (10 µM) for five days determined with Annexin V/PI assay and flow cytometry presented on (**C**) the dot plot (Q1: Early-apoptotic cells—green, Q2; Late-apoptotic cells—red, Q3: Live cells-blue, Q4: Necrotic cells—red) and (**D**) the graph showing the mean ± SEM. Statistical significance was tested using the non-parametric Kruskal–Wallis test with Dunn’s Multiple Comparison post hoc test; * *p* < 0.05; ** *p* < 0.01.

HBFs AS cultured in vitro in response to the TGF-β_1_ develop the myofibroblastic phenotype characterized by an enhanced level of α-SMA incorporated into stress fibers [5,6,7,9,10,12]. To address a possible involvement of p38 MAPK in TGF-β_1_-induced FMT observed in HBF AS populations, we investigated the effects of a specific p38 MAPK inhibitor—SB203580—on the α-SMA level in HBFs. Firstly, we checked the proliferation, viability, and apoptosis of HBFs AS cultured in a serum-free medium containing increasing concentrations of SB203580. We did not observe any significant anti-proliferative effect of the tested compound up to 15 µM concentration in HBFs cultured for five days (Figure 1A). To verify the effect of SB203580 on the viability of HBFs AS, we used the MTT (3-(4,5-dimethylthiazol-2-yl)-2,5-diphenyltetrazolium bromide) assay. Although we observed a slight decrease in the absorbance of the formazan product in HBFs treated with SB203580 for five days, this effect was not significant in samples up to 15 µM concentration (Figure 1B). Since the results of the MTT test (based on the analysis of mitochondrial activity) reflects not only the cytotoxic effect of the investigated compound but also its effect on cell proliferation, we used the Annexin V with propidium iodide (Annexin V/PI) test for additional confirmation of the non-cytotoxic effect of SB203580 on HBF AS populations after five days of culture (Figure 1C,D). We did not observe any significant effect of the tested compound on the induction of apoptosis and necrosis in HBF populations; therefore, in further analyses, we used the SB203580 at a concentration of 10 µM.

**Figure 2:**
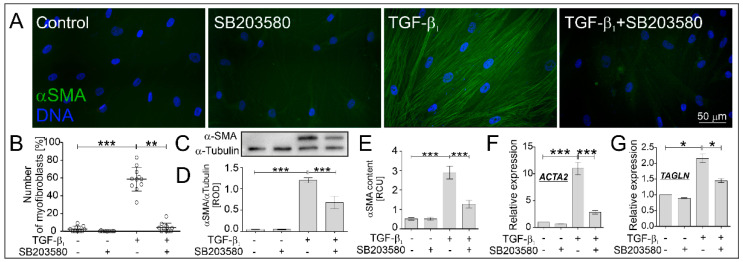
The TGF-β_1_-induced FMT of HBFs AS is significantly inhibited by SB203580. The efficiency of the FMT was checked in HBF AS populations cultured without or with SB203580 (10 μM) in the absence or presence of TGF-β_1_ (5 ng/mL) for five days. (**A**) Representative images of HBFs AS, immunostained for α-SMA (green) and counterstained for DNA (blue), were obtained. Scale bar = 50 µm. (**B**) The percentage of myofibroblasts in HBF AS cultures (*n* = 11) are presented on the graph. The level of α-SMA in HBFs AS (*n* = 3) was determined using (**C**,**D**) Western blot and (**E**) in-cell ELISA. RCU—relative colorimetric units. (**F**,**G**) Relative expression levels of myofibroblast-related genes: *ACTA2* (encoding α-SMA), *TAGLN* (encoding transgelin) in HBFs AS (*n* = 4) cultured for 24 h in conditions described above were presented as a 2^−ΔΔCt^ mean value in relation to control genes (*18S*, *B2M* and *GAPDH*) and control conditions. Statistical significance was tested using the non-parametric Kruskal–Wallis test with Dunn’s Multiple Comparison post hoc test; * *p* < 0.05; ** *p* < 0.01; *** *p* < 0.001.

Further analyses were performed to verify the effect of the p38 MAPK inhibitor on the TGF-β_1_-induced FMT in HBF AS populations (*n* = 11). The FMT potential was expressed as the percentage of myofibroblasts with prominent α-SMA-positive stress fibers. The stimulatory effect of TGF-β_1_ on the myofibroblastic transitions of HBFs AS was strongly inhibited by the p38 MAPK inhibitor—from approx. 60% to approx. 10% (Figure 2A,B). Analyses of α-SMA content at the protein (Figure 2C–E) and gene expression levels (Figure 2F) in HBFs AS treated by SB203580 in the absence or presence of TGF-β_1_ confirmed the observed effect. Furthermore, TGF-β_1_-induced enhancement expression of another myofibroblast-related marker—Sm22 (*TAGLN*)—in HBFs AS was significantly diminished in response to the administration of SB203580 (Figure 2G). These observations demonstrate the inhibitory effect of SB203580 and the possible inhibitory effect of p38 MAPK on TGF-β_1_-induced myofibroblastic transitions of HBFs AS.

**Figure 3:**
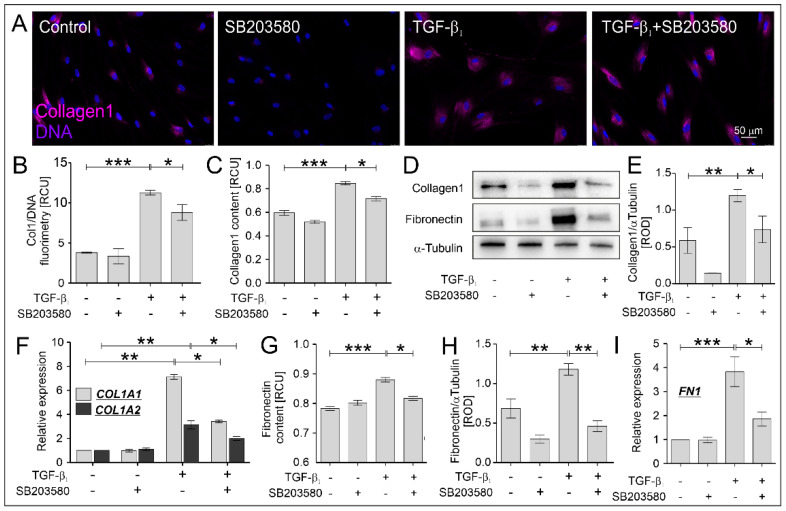
ECM-related myofibroblast markers were significantly reduced by SB203580 in TGF-β_1_-treated HBF AS cultures. HBF AS populations were cultured without or with SB203580 (10 μM) in the absence or presence of TGF-β_1_ (5 ng/mL) for five days. (**A**) Representative images of HBFs AS, immunostained for collagen1 (magenta) and counterstained for DNA (blue), were obtained. Scale bar = 50 µm. The levels of collagen1 in HBFs AS cultures (*n* = 3) were determined (**B**) fluorometrically, (**C**) using in-cell ELISA and (**D**,**E**) Western blot. The levels of fibronectin in the HBF AS cultures (*n* = 3) were determined (**G**) using in-cell ELISA and (**D**,**H**) Western blot (representative membranes are shown). Relative expression levels of (**F**) *COL1A1* (encoding collagen 1A1), *COL1A2* (encoding collagen 1A2) and (**I**) *FN1* (encoding fibronectin) in the HBFs AS (*n* = 4) cultured for one day in conditions described above were presented as a 2^−ΔΔCt^ mean value in relation to control genes (*18S*, *B2M* and *GAPDH*) and control conditions. RCU—relative colorimetric units. Statistical significance was tested using the non-parametric Kruskal–Wallis test with Dunn’s Multiple Comparison post hoc test; * *p* < 0.05; ** *p* < 0.01; *** *p* < 0.001.

It is well known that TGF-β_1_-activated myofibroblasts secrete enhanced levels of ECM proteins, such as collagen 1, fibronectin, and tenascin C, which are described as extracellular markers of FMT [6]. We investigated the effect of the p38 MAPK inhibitor on the collagen 1 and fibronectin levels in HBF AS cultures exposed to TGF-β_1_. Immunofluorescent (Figure 3A,B), immunoenzymatic (Figure 3C) and immunoblotting (Figure 3D,E) studies revealed the strong stimulatory effect of TGF-β_1_ on the collagen1 and fibronectin levels (Figure 3D,G,H) in HBFs AS, which were significantly attenuated by the administration of SB203580. These observations were confirmed by the analyses of myofibroblast-related markers at the gene expression levels. The strong enhancement of *COL1A1*, *COL1A2* and *FN1* expression observed in HBFs AS stimulated by TGF-β_1_ was significantly attenuated in the populations cultured with TGF-β_1_ in the presence of SB203580 (Figure 3F,I). Thus, the administration of SB203580 to the cultures of HBF AS stimulated by TGF-β_1_ led to a significant attenuation of myofibroblast-related markers at the gene and protein levels.

### 2.2. Actin Cytoskeleton Architecture Is Rearranged in TGF-β_1_-Treated HBFs AS after the Administration of SB203580

**Figure 4:**
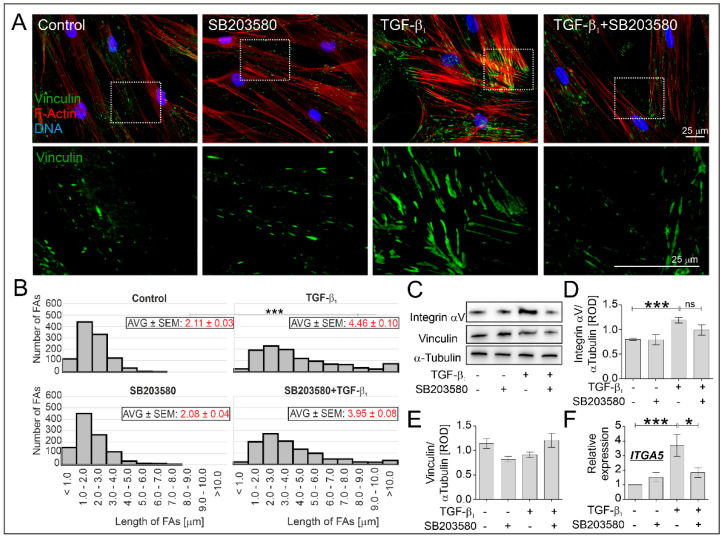
SB203580 affected the vinculin-rich and integrin-mediated adhesion sites in TGF-β_1_-treated HBFs AS. HBF AS populations (*n* = 3) were cultured without or with SB203580 (10 μM) in the absence or presence of TGF-β_1_ (5 ng/mL) for five days. Then, the HBFs AS were immunostained for vinculin (green), counterstained for F-actin (red) and DNA (blue), and the vinculin-rich FAs were imaged using a fluorescence microscope with a TIRF module. (**A**) Representative images of FAs were presented (scale bar = 25 µm), the marked box has been enlarged and presented in the bottom row, (**B**) quantified (average (AVG) length of at least 200 FAs per condition in boxes ± SEM) and grouped by size. (**C**) The protein levels of vinculin and integrin αV in HBF AS cultures (*n* = 3) were determined by Western blot and (**D**,**E**) quantified. Representative membranes were shown. (**F**) Relative expression levels of *ITGA5* (encoding integrin α5) in HBFs AS (*n* = 4) cultured for one day in conditions described above were presented as a 2^−ΔΔCt^ mean value in relation to control genes (*18S*, *B2M* and *GAPDH*) and control conditions. Statistical significance was tested using the non-parametric Kruskal–Wallis test with Dunn’s Multiple Comparison post hoc test; * *p* < 0.05; *** *p* < 0.001; ns—not statistically significant.

Diminished myofibroblast-related markers, such as α-SMA and transgelin detected in TGF-β_1_-treated HBFs AS in response to the administration of SB203580 prompted us to analyze the architecture of the actin cytoskeleton of HBFs. Prominent α-SMA-positive microfilament bundles anchored in focal adhesion sites: vinculin-rich and integrin-related, are associated with the myofibroblastic phenotype of HBFs (Figure 4A). Using fluorescence microscopy with the TIRF (Total Internal Reflection Fluorescence) module, we collected and analyzed the images of vinculin-rich FAs in HBFs AS. Immunofluorescent (Figure 4A) studies showed that FAs observed in the TGF-β_1_-treated HBFs AS were significantly greater than in untreated control cells (Figure 4B). The length of FAs observed in HBFs AS treated by SB203580 in the presence of TGF-β_1_ was slightly reduced, but the total content of vinculin remained unchanged (Figure 4C,E). Analyses of histograms presenting the number of FAs and their length revealed reduced numbers of large FAs (6.0–30.0 µm), termed super mature FAs [31] in response to the administration of SB203580 compared to TGF-β_1_-treated HBFs AS (Figure 4B). Additionally, TGF-β_1_-induced elevated levels of integrin α5 and αV—transmembrane proteins interacting with the ECM component and actin cytoskeleton filaments, were significantly reduced in HBFs AS by SB203580 at the transcript (Figure 4F) and protein (Figure 4C,D) levels, respectively. It suggests that the observed reduction of ECM components, the length of the vinculin-rich FAs and the integrin α5 and αV levels are associated with the diminished TGF-β_1_-induced FMT potential of SB203580-treated HBFs AS and the possible role of p38 MAPK signaling in this process.

### 2.3. SB203580 Diminishes the Levels of Cx43 in TGF-β_1_-Treated HBFs AS in a Smad-Dependent Manner

**Figure 5:**
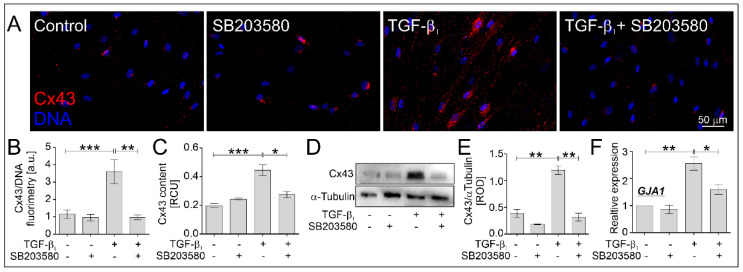
SB203580 significantly reduced the level of Cx43 in TGF-β_1_-treated HBFs AS. HBF AS populations were cultured without or with SB203580 (10 μM) in the absence or presence of TGF-β_1_ (5 ng/mL) for five days. (**A**) Representative images of HBFs AS, immunostained for Cx43 (red) and counterstained for DNA (blue), were obtained. Scale bar = 50 µm. The levels of Cx43 in HBF AS cultures were determined (**B**) fluorometrically (*n* = 11), (**C**) using in-cell ELISA (*n* = 4) and (**D**) Western blot with (**E**) densitometric analysis (*n* = 3). A representative membrane was shown. (**F**) The relative expression of *GJA1* (encoding Cx43) in HBFs AS (*n* = 4) cultured for one day in conditions described above was presented as a 2^−ΔΔCt^ mean value in relation to control genes (*18S*, *B2M* and *GAPDH*) and control conditions. RCU—relative colorimetric units. Statistical significance was tested using the non-parametric Kruskal–Wallis test with Dunn’s Multiple Comparison post hoc test; * *p* < 0.05; ** *p* < 0.01; *** *p* < 0.001.

The effectiveness of TGF-β_1_-induced myofibroblastic transitions of HBF AS populations is associated with the function of connexin 43 (Cx43), a protein involved in the intercellular communications between cells via gap junction channels. Our recent reports indicate a dual function of Cx43 in the regulation of TGF-β_1_-induced FMT [9,12]. In this study, we investigated the effect of SB203580 on the level of Cx43 in TGF-β_1_-treated HBFs AS. According to our previous observations, immunofluorescent (Figure 5A,B), immunoenzymatic (Figure 5C) and immunoblotting (Figure 5D,E) studies revealed the strong stimulatory effect of TGF-β_1_ on the Cx43 level in HBFs AS, which was significantly diminished in response to the administration of SB203580 (Figure 5A–E). Similarly, a decreased expression of Cx43 transcript (*GJA1* gene) in response to the p38 MAPK inhibitor was observed in HBFs AS treated with TGF-β_1_ (Figure 5F). The decreased level of Cx43 correlated with the reduced FMT potential in HBFs AS treated simultaneously with SB203580 and TGF-β_1_ (comp. Figure 2 and Figure 5).

**Figure 6:**
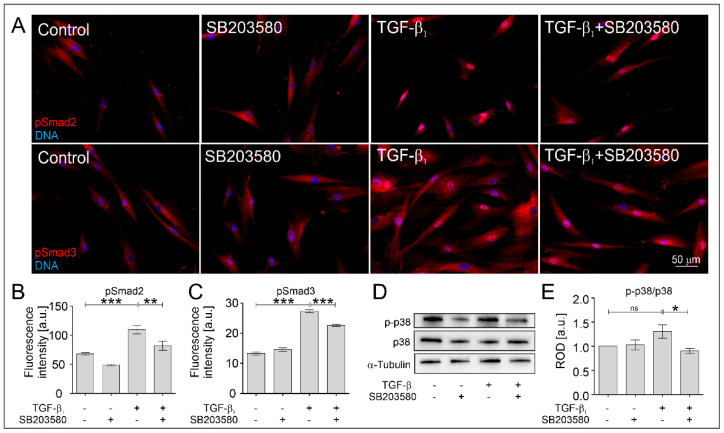
SB203580 affected the TGF-β_1_-induced phosphorylation levels of Smad2 and Smad3 in the cell nuclei area and diminishes the phosphorylation of p38 MAPK in HBFs AS. HBF AS populations were cultured without or with SB203580 (10 μM) in the absence or presence of TGF-β_1_ (5 ng/mL) for one hour. (**A**) Representative images of HBFs AS, immunostained for pSmad2 and pSmad3 (red) and DNA (blue), were presented. Scale bar = 50 µm. (**B**) The mean fluorescence intensity (±SEM) of pSmad2 and (**C**) pSmad3 in the cell nuclei area was measured using ImageJ (*n* = 3; at least 100 cells per condition). (**D**) The levels of p-p38 and p38 MAPK in HBF AS cultures (*n* = 3) were determined using Western blot and quantified in (**E**). Representative membranes were shown. The statistical significance of the differences between conditions was determined by the non-parametric Kruskal–Wallis test with Dunn’s Multiple Comparison post hoc test, * *p* < 0.05; ** *p* < 0.01; *** *p* < 0.001; ns—not statistically significant.

According to our previous reports [9,12] Cx43 interacts with Smad2 proteins and regulates the TGF-β_1_-activated Smad2/3-dependent signaling pathway. In the next step, we investigated the effect of SB203580 on the TGF-β-activated Smad2/3 signaling. Immunofluorescent studies revealed that the TGF-β_1_-induced nuclear translocation of phosphorylated Smad2 proteins was significantly diminished by SB203580 (Figure 6A, quantified in B). Furthermore, the TGF-β_1_-induced phosphorylation level of Smad3 in the cell nuclei area was also diminished in response to the administration of SB203580 (Figure 6A, quantified in C). Due to the fact that TGF-β_1_ has a pleiotropic effect on the activation of different signaling pathways, including MAPK cascades, we also investigated the levels of phosphorylated p38 in HBFs AS after the administration of TGF-β_1_ without or with the MAPK inhibitor. SB203580 diminished the p-p38 level in both un-treated and TGF-β_1_-treated HBFs (Figure 6D,E). This suggests that the observed reduction of myofibroblast-associated proteins (α-SMA and Cx43) in response to the administration of SB203580 in TGF-β_1_-treated HBFs AS is at least partially dependent on the attenuation of the activity of the p38 MAPK signaling cascade in cooperation with Smad2/3 pathway impairment.

## 3. Discussion

The phenomenon of fibroblast-to-myofibroblast transitions observed during the progression of fibrosis-associated diseases is intensively studied to clarify its mechanisms, which can be varied in different tissues. Under profibrotic pathological conditions, the populations of myofibroblasts remain constitutively active and in consequence, lead to the overproduction of ECM proteins, tissue malformations and functional impairment. This is documented in the skin [32], liver [33], heart [34] and kidney fibrosis [35], lung diseases as a chronic obstructive pulmonary disease (COPD) [36], idiopathic pulmonary fibrosis (IPF) [37] and also in subepithelial fibrosis in asthma [6]. Routinely used therapies for asthma primarily affect the inflammatory processes in the airways and the symptoms of asthma but are insufficient or ineffective in case of the development of bronchial subepithelial fibrosis [6], which can be driven also in an inflammation-independent manner [38,39,40]. For this reason, the excessive activity of a large population of myofibroblasts as well as the mechanisms of the bronchial subepithelial fibrosis progression leading to the functional impairment of lung tissue in asthmatics remain a challenge for contemporary medicine and cell biology [41] and are a very interesting and highly important target for new therapeutic strategies.

The intensive research conducted so far has not yet provided sufficient knowledge about the FMT mechanisms; therefore, there is a need to continue and expand this research. It is well documented that phenotypic transitions of HBFs in asthmatics can be induced by several profibrotic and proinflammatory cytokines and growth factors, but TGF-β_1_ is known as the most potent activator of FMT [6]. The effectiveness of this process is dependent not only on the presence of humoral factors but also on mechanical stimuli received by HBFs from the bronchi microenvironment [6]. Excessive deposits of ECM proteins are observed in the airway tract of most patients with diagnosed asthma, which favors the phenotypic shifts of bronchial fibroblasts [41]. Our previous studies show the enhanced FMT potential of TGF-β_1_-treated HBFs from asthmatic donors in comparison to non-asthmatic ones [7,8,9,10,11]. We hypothesize that increased FMT in HBFs AS is caused by the different responses of these cells to TGF-β_1_ stimulation, which has been partially confirmed by previous studies [6,7,9,10,11]. Chronic exposition of HBFs at relatively high doses of TGF-β_1_ leads to the start of the transcription of FMT-related genes by the activation of multiple intracellular signaling pathways, but the primarily canonical Smad signaling is intensively investigated [42]. The involvement of TGF-β_1_-induced non-canonical MAPK signaling (e.g., ERK-, c-Jun-, or p38-dependent) in the progression of cardiac, liver, kidney, or lung fibrosis was described previously [14,21,23,25,26,27]. On the other hand, the impact of p38 MAPK was described in the other processes (anti-inflammatory cytokine releasing from, e.g., alveolar macrophage or bronchial epithelium) involved in the progression of asthma and COPD [29,43,44,45]. However, little is known about the role of p38 MAPK in the progression of subepithelial fibrosis observed in asthmatic bronchi. It has become a starting point to consider and verify the role of p38 MAPK in the TGF-β_1_-induced FMT in HBF AS populations in culture.

The experimental model used in this study, based on the HBF cultures, was established from bronchoscopy biopsies derived from asthmatic patients. Our previous reports confirmed its usefulness for basic research of TGF-β_1_-induced FMT and its mechanisms [9,10,11,12]. The use of HBFs isolated from asthmatic patients instead of cell lines as MRC5 or IMR-90 is more favorable for these studies due to the mimics of the microenvironment of asthmatic bronchi and reveals unique features that predispose these cells to increased FMT [6].

In this study, we show that the inhibition of p38 MAPK by a chemical inhibitor (SB203580) significantly attenuates the TGF-β_1_-induced FMT potential in HBFs AS, measured by the percentage of myofibroblasts in the tested HBF population, which is also associated with decreased levels of myofibroblast-related genes (*ACTA2*, *TAGLN*) and proteins, such as α-SMA. We also show that the strong enhancement of collagen 1 and fibronectin expression (the main ECM-related markers of myofibroblasts) observed in TGF-β_1_-treated HBFs AS was significantly decreased after the administration of SB203580. Similar observations were described previously in the populations of human tenon fibroblasts [46], human Graves’ orbital fibroblasts [47], nasal polyp-derived fibroblasts [48], in bradykinin-induced FMT in HBFs [49], in cardiac fibroblasts [25,50], and in the rat model of kidney fibrosis [51]. Several studies indicate that the administration of p38 MAPK inhibitors ameliorates pulmonary fibrosis by the inhibition of the hydroxyproline content in whole lung tissue lysates in bleomycin-induced murine or Lewis rat model [52,53]. Furthermore, the p38 MAPK inhibition by SD-282 reduced inflammation, hyperplasia of airway epithelium, goblet cell metaplasia and mucus secretion and subepithelial fibrosis was observed in the transgenic mouse model of asthma [54]. The beneficial effect of the p38 MAPK inhibitor on the structure of the respiratory tract was associated with an improvement of lung function and the severity of asthma in this model [54]. These findings clearly indicate the strong therapeutic potential of p38 MAPK inhibitors in the reduction of both inflammatory components and fibrosis-related events observed in, in vivo models of asthma [52,53,54].

Our recent reports documented that Cx43 plays a key role in the regulation of TGF-β_1_-induced AS HBF phenotypic transitions in both a gap junction intercellular coupling (GJIC)-dependent and -independent manner [9]. Cx43 interacts with different proteins as Smads or tubulins via the carboxyl tail in a GJIC-independent manner and regulates FMT efficiency [9]. In this study, we show for the first time the strong effect of p38 MAPK inhibition on the levels of Cx43 upregulated by TGF-β_1_ in HBFs AS. The inhibitory effect of SB203580 on the relatively high Cx43 levels was also described in corneal [55,56] and cardiac fibroblasts [57]. Upregulation of myofibroblast-related protein levels induced by TGF-β_1_ (e.g., α-SMA or Cx43) are strongly associated with the enhanced phosphorylation and transcriptional activity of Smad proteins [9,12]. In our study, we observed that levels of phosphorylated Smad2 and Smad3 in the cell nuclei area are upregulated by TGF-β_1_ in HBFs AS, which were significantly attenuated by the p38 MAPK inhibitor. The inhibitory effect of SB203580 on the Smad2 and/or Smad3 phosphorylation levels were described in the TGF-β_1_-treated corneal fibroblasts [58], HepG2 cells [59], cardiac fibroblasts [50], hepatic stellate cells [60] and trabecular meshwork cells [61]. Although our results do not clearly indicate the mechanism of TGF-β_1_-induced FMT attenuation observed in the HBF AS populations in response to the administration of p38 MAPK, we suggest the occurrence of similar processes leading to the FMT reduction as described in cardiac fibroblast. Liu et al. [50] showed that the inhibition of Smad2 phosphorylation by SB431542 led to the attenuation of p38 phosphorylation, and the p38 MAPK inhibitor—SB203580—reduced Smad2 phosphorylation. This suggests cross-talk or interactions between these pathways, but the mechanisms and the biological relevance require additional studies [50]. The presence of interactions between the p38 MAPK and Smad3 signaling was also described in liver fibrogenesis, especially during the differentiation of hepatic stellate cells induced by TGF-β_1_ [60]. Furukawa et al. indicated that the TGF-β_1_-activated p38 MAPK signaling enhanced the phosphorylation level of Smad3 and led to extracellular matrix production and secretion. By using highly specific antibodies, they showed that the application of the p38 MAPK inhibitor (SB203580) suppresses the phosphorylation of Smad3 at the linker region, leading to the suppression of liver fibrotic changes [60]. Thus, we suggest that down-regulation of profibrotic genes and myofibroblast-related proteins following the inhibition of p38 MAPK signaling in TGF-β_1_-treated HBFs AS may be at least partially the result of the inhibition of Smad transcriptional factors, phosphorylation and cross-reactivity with p38 MAPK signaling. However, this hypothesis requires additional studies.

According to the current knowledge, FMT can be regulated by mechanical factors and signals from extracellular matrix components [6]. Profibrotic changes of ECM properties and composition affect the actin cytoskeleton by the focal adhesion sites which act as signal transmitters. Our previous study shows that non-stimulated HBFs AS are characterized by a highly developed actin cytoskeleton with numerous thick and aligned ‘ventral’ stress fibers accompanied by enlarged focal adhesions when compared with HBFs derived from non-asthmatic patients. The altered actin cytoskeleton architecture of HBFs AS correlate with their higher elastic modulus and increased predilection to TGF-β_1_-induced FMT [62]. In this study, we show that the inhibition of p38 MAPK by SB203580 affects the maturation of super mature vinculin-enriched FAs but significantly attenuates the integrin α5 and αV expression levels. Previously, it was shown that the inhibition of p38 MAPK signaling resulted in the abrogation of stretch-induced cytoskeletal reorganization in mouse wild type-derived and human uterosacral ligament fibroblasts [63,64]. On the other hand, other studies report that the status of FA maturation is associated with the ECM composition as well as with the actin cytoskeleton architecture [65,66,67]. The disturbed maturation of vinculin-rich FAs and decreased levels of components of integrin-mediated FAs concomitantly with the changes in ECM composition caused by p38 MAPK inhibition can also affect the actin cytoskeleton architecture in TGF-β_1_-treated HBFs AS. Disturbed maturation of adhesion sites in HBFs AS treated by the p38 MAPK inhibitor can affect the mechanical tension and makes it difficult to stress fibers formation and α-SMA incorporation into microfilament bundles, which causes the reduced FMT potential in HBFs AS. 

Finally, in this study, we show for the first time the strong effect of the p38 MAPK inhibitor (SB203580) on the FMT potential of TGF-β_1_-stimulated HBF populations derived from asthmatic patients. This phenomenon is associated with the strong attenuation (up to control levels) of myofibroblasts-related markers, such as α-SMA, collagen I, fibronectin and Cx43 in TGF-β_1_-treated HBFs AS. Mechanistically, the strong effect of p38 MAPK inhibition by SB203580 affects the impairment of TGF-β_1_/Smad signaling, but not up to the control level. However, such a low level of myofibroblastic markers in HBFs AS stimulated by TGF-β_1_ in the presence of SB203580 indicates the additional mechanisms of p38 MAPK inhibition on the FMT potential. Perhaps disturbances in the activation of the TGF-β/p38 and TGF-β/Smad2/3 signaling pathways and possible interactions between them in conjunction with the physiological GJIC-independent function of Cx43 lead to the strong attenuation of the profibrotic potential of HBFs AS exposed to TGF-β_1_. The other possible mechanism of FMT attenuation in HBFs AS can be similar to this described in epithelial or osteocyte-like cells where Cx43 interacts directly with integrin α5 [68,69] and indirectly with ECM components, e.g., fibronectin or collagen I [70,71,72]. On the other hand, Cx43 can play a role in signal mechanotransduction, which was documented in cardiac fibrosis [73]. The existence of direct interrelations between Cx43, integrin α5 and ECM components can play a role in the regulation of FMT observed in HBFs AS but cannot be unequivocally indicated or excluded on the basis of the available data. It requires additional studies to elucidate the precise mechanism of pleiotropic action of the p38 MAPK inhibitor on the TGF-β_1_-induced myofibroblastic transition of HBFs AS. However, in our opinion, the targeting of p38 MAPK activity, especially through the use of p38 MAPK inhibitors, such as SB203580, can be considered as a possible pharmacological target for the inhibition of TGF-β_1_-induced myofibroblast transitions of HBFs during subepithelial fibrosis associated with asthma.

## 4. Materials and Methods

### 4.1. Cell Cultures

This study was performed on the populations of HBFs isolated from bronchoscopy biopsies derived from 11 patients diagnosed with asthma (3–5 according to the Global Initiative for Asthma classification) and established as described previously [10]. The study group consisted of seven females and four males aged 45.73 ± 16.63 years, with reduced average of forced expiratory volume in one second (FEV1)%: 72.16 ± 13.36. The average duration of asthma is 13 ± 11.86 years and the average age of diagnosis was 39.1 ± 15.1 years. All patients were treated in the Department of Medicine of the Jagiellonian University Medical College and remained in stable clinical conditions. The study was approved by the University’s Ethics Committee (KBET Decision No. 122.6120.16.2016; 28 January 2016). HBFs characterized by vimentin-positive and desmin-negative staining were cultured in a complete medium: Dulbecco’s modified Eagle’s medium with high glucose (DMEM HG, Sigma-Aldrich, St. Louis, MO, USA) supplemented with 10% fetal bovine serum (FBS, Gibco, Thermo Fisher Scientific, Waltham, MA, USA) and a penicillin/streptomycin solution (P4333; Sigma-Aldrich, St. Louis, MO, USA) in standard conditions (37 °C and 5% CO_2_). For the experiments (unless otherwise noted), HBF populations (between the 5th and 20th passages) were seeded at a density of 5000 cells/cm^2^ and cultured in a complete medium for 24 h. Then cells were cultured in a serum-free medium—DMEM HG containing 0.1% bovine serum albumin (BSA, Sigma-Aldrich, St. Louis, MO, USA) in the absence or presence of a p38 inhibitor (SB203580; 0–20 μM; Sigma-Aldrich, St. Louis, MO, USA) without or with human recombinant TGF-β_1_ (5 ng/mL; Corning, NY, USA), as the inductor of phenotypic shifts in HBF populations.

### 4.2. Analysis of Viability, Proliferation and Apoptosis of HBFs

HBFs were cultured in 96-well plates for 24 h in a complete medium, which was changed for a serum-free medium without or with SB203580 (0–20 μM) for one, three, or five days. Then, the viability and proliferation rate of the HBFs were determined using MTT assay or crystal violet (CV) assay (described previously [10]), respectively. The absorbance of the samples was measured using a microplate reader (Multiskan FC; Thermo Fisher Scientific, Waltham, MA, USA) at 570 nm (MTT assay) and 540 nm (CV assay). Three independent experiments were performed for each condition. Apoptosis in HBFs treated by SB203580 (10 µM) for five days was determined using the Annexin V/PI Assay Kit (Biotium, Fremont, CA, USA). For each sample, 3 × 10^4^ of the cells were collected within 1 h of staining on an LSRII flow cytometer (BD Biosciences, San Jose, CA, USA). Instrument settings were determined using control and staurosporine-treated samples (1 μM), both stained and unstained. Data were analyzed using FACS Diva software (v7.0; BD Biosciences, San Jose, CA, USA). All events were gated to exclude cell debris (SSC-A vs. FSC-A) and cell doublets (FSC-A vs. FSC-W). Single cells were assigned to four quadrants based on annexin V (AV) and propidium iodide (PI) fluorescence in the following manner: (1) AV-negative, PI-negative cells to the population of healthy cells, (2) AV-positive, PI-negative cells to the population of early-apoptotic cells, (3) AV-negative, PI-positive cells to the population of necrotic cells, (4) AV-positive, PI-positive cells to the population of late-apoptotic cells.

### 4.3. Immunofluorescence Studies

Cells were seeded on glass coverslips in a 12-well plate in a complete medium. After 24 h the medium was changed to a serum-free medium without or with SB203580 (10 μM) in the absence or presence of TGF-β_1_ (5 ng/mL; Corning, NY, USA). After five days of incubation, the HBFs were fixed with 3.7% formaldehyde/PBS for 15 min then permeabilized with 0.1% Triton-X100/PBS, and non-specific binding sites were blocked with 1% BSA in PBS. Samples were incubated overnight with primary antibodies (mouse monoclonal IgG anti-α-SMA; rabbit polyclonal IgG anti-connexin43; mouse monoclonal IgG anti-vinculin; rabbit polyclonal IgG anti-collagen1; all from Sigma-Aldrich, St. Louis, MO, USA; rabbit polyclonal IgG anti-pSmad2, rabbit polyclonal IgG anti-pSmad3, from Cell Signaling Technology, Danvers, MA, USA) and then with appropriate secondary antibodies: goat anti-mouse or goat anti-rabbit antibodies conjugated with Alexa Fluor 488 or Alexa Fluor 546 (Life Technologies, Thermo Fisher Scientific, Waltham, MA, USA). Samples were counterstained with Hoechst 33258 (1 µg/mL; Sigma-Aldrich, St. Louis, MO, USA) for DNA visualization and/or phalloidin conjugated with Alexa Fluor 546 (Life Technologies, Thermo Fisher Scientific, Waltham, MA, USA) for the detection of F-actin bundles. The images were collected using a Leica fluorescence microscope DMI6000B with LasX software (v3.7.4; Leica Microsystems GmbH, Wetzlar, Germany) equipped with a TIRF (Total Internal Reflection Fluorescence) module. The lengths of focal adhesions from images captured by TIRF microscopy were measured with ImageJ freeware (NIH, Bethesda, MD, USA). Fluorimetry was performed using collected images captured with identical excitation and exposure settings. The results were presented as an average measured intensity of connexin43 or collagen1 fluorescence in relation to DNA fluorescence intensity [9] or the fluorescence of pSmad2 and pSmad3 from the nuclear area using ImageJ freeware (NIH, Bethesda, MD, USA).

### 4.4. In-Cell ELISA Assay

Cells were cultured in 96-well plates for 24 h in a complete medium, which was changed for a serum-free medium without or with SB203580 (10 µM) alone or in combination with TGF-β_1_ (5 ng/mL; Corning, NY, USA). After five days of culture, the HBFs were fixed and permeabilized with ice-cold methanol and blocked with a 1% BSA/PBS solution with 0.1% Tween20/PBS. The HBFs were incubated overnight at 4 °C with primary antibodies in a 1% BSA/PBS solution (mouse monoclonal anti-α-SMA; rabbit polyclonal anti-fibronectin; rabbit polyclonal anti-Cx43; rabbit polyclonal anti-collagen1; all from Sigma-Aldrich, St. Louis, MO, USA). After triple rinsing, the plates were incubated with goat anti-mouse or goat anti-rabbit secondary antibodies conjugated with horseradish peroxidase (HRP) in a 1% BSA/PBS solution at room temperature for 1 h. Colorimetric reactions were induced by the addition of tetramethylbenzidine (Sigma-Aldrich, St. Louis, MO, USA) and stopped using 1N HCl/H_2_O. Absorbances were measured using MultiskanFC at 450 nm.

### 4.5. Immunoblotting

The preparation of protein lysates and the measurement of protein content and western blots were performed according to the protocols described previously [9,12]. The membranes were incubated overnight at 4°C with primary antibodies (mouse monoclonal IgG anti-α-SMA, mouse monoclonal IgG anti-vinculin, rabbit polyclonal IgG anti-fibronectin, rabbit polyclonal IgG anti-collagen 1, rabbit polyclonal IgG anti-connexin 43 and mouse monoclonal IgG anti-α-tubulin (Sigma-Aldrich, St. Louis, MO, USA); rabbit polyclonal IgG anti-integrin alpha 5, rabbit polyclonal IgG anti-p38, and rabbit polyclonal IgG anti-p-p38 (Cell Signaling Technology, Danvers, MA, USA)), diluted in a 1% BSA solution in Tris-buffered saline with Tween20 (TBST). Then, the membranes were incubated with secondary antibodies (goat anti-mouse or goat anti-rabbit) conjugated with HRP (Thermo Fisher Scientific, Waltham, MA, USA). Luminata Crescendo Western HRP Substrate (Merck Millipore, Burlington, MA, USA) and a chemiluminescence imaging system ChemiDoc XRS + (Bio-Rad, Hercules, CA, USA) were used for band detection. Relative optical densities (RODs) were quantified with ImageJ software (v2.1.0/1.53c).

### 4.6. Quantitative Real-Time PCR

The HBFs (1 × 10^4^ per cm^2^) were cultured in 6-well plates for 24 h in a complete medium, which was changed for a serum-free medium without or with SB203580 (10 µM) alone or in combination with TGF-β_1_ (5 ng/mL; Corning, NY, USA). After 24 h, the HBFs were lysed and total RNA was isolated from the HBFs using the GeneMATRIX Universal RNA/miRNA Purification Kit (EURx, Gdansk, Poland) and then reverse transcribed to cDNA with the NG dART RT-PCR Kit (EURx, Gdansk, Poland) in a C1000 Touch Thermal Cycler (Bio-Rad, Hercules, CA, USA) according to the manufacturer’s protocol. The expression levels of selected human genes (*ACTA2*, *TAGLN*, *GJA1*, *COL1A1*, *COL1A2*, *FN1*, *ITGA5*, *18S*, *B2M*, *GAPDH*) were specified by real-time PCR assay using SYBR Green PCR Master Mix (Applied Biosystems, Waltham, MA, USA) and specific primer sets (sequences listed in Table 1; all from Genomed, Warsaw, Poland) using 7500 Fast System (Applied Biosystems, Waltham, MA, USA). The relative expression of selected genes was estimated using the quantification threshold value recalculated against the average of *GAPDH*, *18S* and *B2M* transcripts by the ΔCT method [ΔCT refers to CT_TESTED GENE_—CT_GAPDH_]. The results were presented in relation to the control as a 2^−ΔΔCt^ mean value [74].

### 4.7. Statistical Analysis

The values were presented as the mean ± SEM. The normality of distribution was estimated using the Shapiro-Wilk test. Statistical significances were determined using the non-parametric Kruskal–Wallis test with Dunn’s Multiple Comparison post hoc test. * *p* < 0.05; ** *p* < 0.01; *** *p* < 0.001 was considered statistically significant.

## Figures and Tables

**Table 1:** Primer sets used for real-time PCR.

Gene	Protein	Sequence 5′–3′	
		F’	R’
*ACTA2*	α-SMA	CTGTTCCAGCCATCCTTCAT	CCGTGATCTCCTTCTGCATT
*B2M*	β2-microglobulin	AATGCGGCATCTTCAAACCT	TGACTTTGTCACAGCCCAAGATA
*COL1A1*	Collagen 1A1	CTTTGCATTCATCTCTCAAACTTAGTTTT	CCCCGCATGGGTCTTCA
*COL1A2*	Collagen 1A2	TGCTGCTGGTCAACCTGGTGC	ACTTCCAGCAGGACCGGGGG
*FN1*	Fibronectin	TGTGGTTGCCTTGCACGAT	GCTTGTGGGTGTGACCTGAGT
*GAPDH*	Glyceraldehyde-3-phosphate dehydrogenase	GAAGGTGAAGGTCGGAGT	GAAGATGGTGATGGGATTTC
*GJA1*	Connexin 43	AGGAGTTCAATCACTTGGCG	GAGTTTGCCTAAGGCGCTC
*ITGA5*	Integrin αV	CCGTGTGGTTTTAGGTGGAC	TTGATCAGGTACTCGGGGTAA
*TAGLN*	Transgelin	CGTGGAGATCCCAACTGGTT	AAGGCCAATGACATGCTTTCC
*18S*	18S rRNA	GTAACCCGTTGAACCCCATT	CCATCCAATCGGTAGTAGCG

## Data Availability

The data presented in this study are available on request from the corresponding author.
